# Indocyanine Green Fluorescence Guided Surgery in Colorectal Surgery

**DOI:** 10.3390/jcm12020494

**Published:** 2023-01-07

**Authors:** Zoe Garoufalia, Steven D. Wexner

**Affiliations:** Ellen Leifer Shulman and Steven Shulman Digestive Disease Center, Cleveland Clinic Florida, Weston, FL 33331, USA

**Keywords:** colon, rectal, colorectal, indocyanine green (ICG), fluorescence, surgery

## Abstract

Background: Indocyanine green (ICG) imaging has been increasingly used for intraoperative guidance in colorectal surgery over the past decade. The aim of this study was to review and organize, according to different type of use, all available literature on ICG guided colorectal surgery and highlight areas in need of further research and discuss future perspectives. Methods: PubMed, Scopus, and Google Scholar databases were searched systematically through November 2022 for all available studies on fluorescence-guided surgery in colorectal surgery. Results: Available studies described ICG use in colorectal surgery for perfusion assessment, ureteral and urethral assessment, lymphatic mapping, and hepatic and peritoneal metastases assessment. Although the level of evidence is low, results are promising, especially in the role of ICG in reducing anastomotic leaks. Conclusions: ICG imaging is a safe and relatively cheap imaging modality in colorectal surgery, especially for perfusion assessment. Work is underway regarding its use in lymphatic mapping, ureter identification, and the assessment of intraperitoneal metastatic disease.

## 1. Introduction

Indocyanine green (ICG) is a fluorescent water-soluble dye that binds with plasma proteins, especially lipoproteins, and demonstrates fluorescent properties in the near-infrared (NIR) spectrum (750–950 nm). It was developed by Kodak Laboratories in 1955 for near-infrared photography and received FDA approval for human use in 1959 [1]. Since then, it has been used as an imaging method in several medical applications, including vascular, endocrine, hepatopancreaticobiliary, gynecologic, plastic, urologic, and colorectal surgery [2].

**Figure 1:**
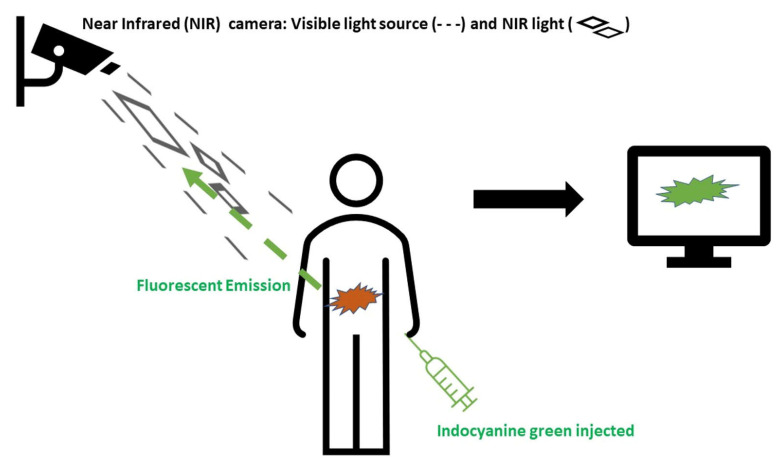
The basics of indocyanine green (ICG) imaging in colorectal surgery. ICG is injected (method of administration varies depending on the target). The ICG molecules are excited by the light source and demonstrate fluorescent properties in the near-infrared spectrum (750–950 nm). This signal is combined with real time image and transferred on the screen.

ICG is metabolized solely through the liver and excreted into the bile, and its half-life depends on liver function (typically 3–4 min) [2]. When it is not directly injected into the bloodstream, it is drained through the lymphatic network. The time required to reach the nearest lymph node is approximately 15 min [3]. ICG has been proven safe with dosages between 0.1–0.5 mg/mL/kg with only the relative contraindications of iodine or shellfish allergy. Depending on the target structure/organ that needs to be visualized, it can be administered through various routes, including intravenously, submucosally or into the ureteral system (Figure 1).

The different ICG applications in colorectal surgery are substantial and still evolving. ICG has been used in colorectal surgeries for perfusion assessment, intraoperative ureteral visualization, sentinel node identification, and lymphatic drainage visualization. Another described use is for the localization and intraoperative assessment of peritoneal and hepatic metastases. However, the evidence provided by published studies is poor. Therefore, the aim of this review was to review and organize, according to different type of use, the available published literature related to ICG-guided colorectal surgery, highlight areas in need of further research, and discuss future perspectives.

## 2. Methods

PubMed, Scopus, and Google Scholar databases were searched systematically through November 2022. The terms ‘colorectal’, ‘colon’, ‘rectal’, ‘indocyanine green’, ‘ICG’, and ‘fluorescence’ were used combined with the Boolean operators AND/OR in order to identify all available studies on fluorescence-guided surgery in colorectal surgery. The reference lists of retrieved articles were also screened for further eligible studies. After removal of duplicates, the abstract list generated by the above search was independently screened by the authors for potentially relevant studies. All English-text available studies involving humans (either cadaveric models or alive patients) on fluorescent guided colorectal surgery were retrieved and synthetized in a narrative manner to describe current applications of this technique as well as its future perspectives.

We did not seek ethics approval as this type of study, a literature review, does not require approval since no patient data were accessed or analyzed.

## 3. Current Uses of ICG in Colorectal Surgery

### 3.1. Perfusion Assessment

#### 3.1.1. Anastomotic and Bowel Perfusion Assessment

Anastomotic leak (AL) remains a major dreaded colorectal surgery complication. AL rates vary by type of surgery with an incidence of up to 19% [4]. Multiple factors contribute to AL; nevertheless, a well perfused and tension-free anastomosis is necessary for an uneventful anastomotic healing process. ICG provides the ability to objectively intraoperatively assess anastomotic perfusion.

**Figure 2:**
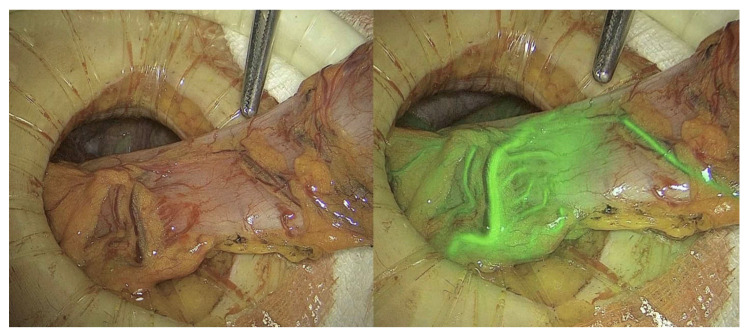
Bowel perfusion assessment using ICG. Courtesy of International Society for Fluorescence Guided Surgery (ISFGS).

Several case series and cohort studies have demonstrated a reduced AL rate with the use of intraoperative ICG for the assessment of anastomotic perfusion, although not always statistically significant [5,6,7,8,9]. The first randomized trial (RCT) was published in 2020 by De Nardi et al. [10] and 252 patients. Although the authors concluded that ICG was safe and not time-consuming, it failed to show significantly lower AL rates (5% in the ICG group versus 9% in the control group). At the same time, another single center RCT was published by Alekseev et al. [11] that showed a significant reduction in AL rates of low (4–8 cm from the anal verge) stapled colorectal anastomoses (14.5% vs. 25.7%) and a change in resection margin in 20% of patients. Following these contradictory results, another randomized control trial was launched in the USA. Unfortunately, due to the low recruitment rate it was deemed underpowered and thus terminated early [12]. Two recent meta-analyses [13,14] concluded that the intraoperative use of ICG for perfusion assessment was associated with significantly lower odds for AL (OR 0.52, 95% CI 0.304–0.98, I2 = 0). One of these metanalyses [14] reported that the weighted mean rate of change in the surgical plan due to ICG imaging was 9.6% (95% CI 7.3–11.8). Currently, another phase III multicenter randomized controlled trial is being launched (NCT04712032; NL7502) [15] aiming to provide high quality evidence regarding the role of ICG in the reduction of clinically relevant ALs. The current literature reports that ICG assessment is a safe and rapid means of assessing anastomotic perfusion/bowel perfusion (Figure 2) and may result in reduction of AL. One of the main drawbacks of this method is the fact that assessment is subjective. Therefore, recent studies [16,17,18,19] have tried to address this issue with software assessing the ICG saturation. One study [16] reported the optimal distance of the camera from the colon to assess perfusion (5 cm) and the optimal time for assessment (1.5–3.5 min after intravenous infusion), indicating some progress in this area. Still, more research is required to identify the optimal software for validated, quantifiable, and reproducible perfusion assessment. Finally, a recent cost analysis [20] of the routine use of ICG for anastomotic perfusion assessment, using the assumption that the cost of AL is >USD 5616.29 and the cost of ICG-FA is <USD 634.44 [odds ratio (OR) for AL reduction with ICG use: 0.46], found that routine use of ICG imaging in colorectal surgery is cost-effective. Video S1 demonstrates the intraoperative use of ICG for bowel perfusion assessment.

#### 3.1.2. Perfusion Assessment in Pedicled Omentoplasty

In 2019, Slooter et al. reported a pilot study for assessing omental perfusion during omentoplasty in salvage surgery for pelvic sepsis [21]. After establishing the feasibility of this technique, the same group went on to demonstrate the possible beneficial role in a subsequent cohort study [22]. They reported a much lower perineal non-healing rate in the group where ICG was utilized (22%) versus the controls (42%).

#### 3.1.3. Perfusion Assessment in Gracilis Muscle Interposition

Graciloplasty is a technique that can be utilized to treat complex perianal fistulae [23]. Nevertheless, a well-perfused gracilis flap is of utmost importance for the success of this technique. A recent study by Lobbes et al. [24] reported a software-based approach for gracilis perfusion assessment, pointing to objective indicators for flap perfusion. This technique may be further developed and utilized in the future to optimize the outcome of gracilis muscle interposition.

#### 3.1.4. Perfusion Assessment in Anal Advancement Flaps

Endorectal advancement flaps are used to treat complex perianal fistula repair; success depends on the viability of the anal flap to avoid flap dehiscence or recurrent fistula. Turner et al. [25] published a retrospective case series of six patients undergoing advancement flaps for perianal fistula. They reported a high rate (71.4%) of change in the surgical plan based on the results of intraoperative ICG perfusion assessment of the flap. This technology may be further developed in the future in terms of the quantification of the result to optimize the outcomes of this technique.

#### 3.1.5. Perfusion Assessment in Ileal Pouch-Anal Anastomosis (IPAA)

Good perfusion is of utmost importance during construction of the anastomosis for IPAA. In 2021, Joosten et al. [26] published the first technical note on utilizing ICG technology to enable lengthening maneuvers. Following this, Freund et al. [27] confirmed the utility of ICG imaging in complex IPAA surgery requiring lengthening maneuvers in their patient series. Similarly, Slooter et al. [28] demonstrated the use of ICG in IPAA surgery, focusing on the time needed between ICG infusion and the assessment of pouch perfusion. They concluded that due to vascular ligation in lengthening maneuvers, a longer time interval might be needed between ICG infusion and the first signal. However, can this assessment be quantifiable? How can one assess the ICG signal with certainty and objectively? Lobbes et al. [29] sought to address this issue using novel software to assess the ICG signal for pouch perfusion in order to make this assessment quantifiable and comparable. Although the available literature supports the use of ICG in IPAA surgery, the level of evidence is still low.

### 3.2. Vital Structures Assessment

#### 3.2.1. Intraoperative Ureteral Assessment

**Figure 3:**
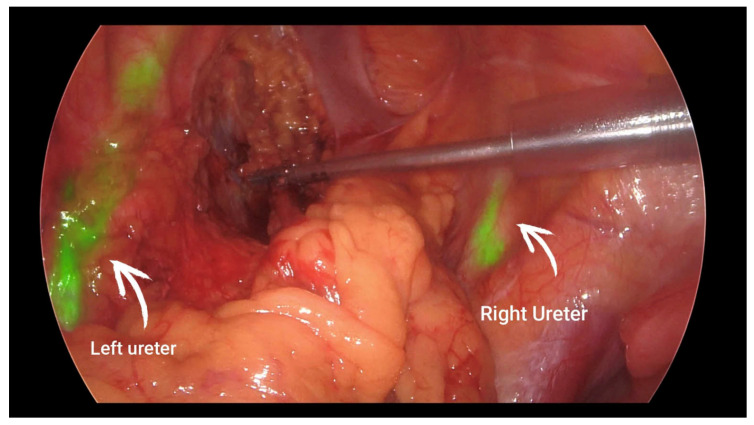
Ureter identification during pelvic dissection. Steven D. Wexner personal archive.

Ureteral injury is a devastating complication in colorectal surgery. The ureters are retroperitoneal structures that can be easily damaged during the dissection of the colon or rectum, especially in complicated surgeries, causing extensive inflammation and the need for re-operations. There are reports in the literature regarding ICG imaging utilization for intraoperative ureteral identification [30,31,32,33,34,35,36]. This method involves cystoscopic ureteral stenting and the injection of ICG dye through the stent into each ureter. Although the exact quantity of ICG instilled varies, the majority of studies report using 5 mL of 2.5 mg/mL ICG solution in each ureter. This dosage allows the ureters to glow green intraoperatively and are thus easier to identify throughout the surgery, especially in minimally invasive surgery where haptic feedback is unavailable. A recent systematic review of this technique [37] concluded that ICG ureter visualization (Figure 3) is safe and effective in minimally invasive colorectal surgery, with one caveat: this technique still entails ureteral stenting and its potential complications. The authors noted that current research is focusing on experimental dyes that could potentially combine imaging properties with renal excretion to minimize the adverse effects of ureteral stenting. In this scope, experimental novel fluorescent dyes are currently under development, similar to those reported by Dip et al. [38] and Mahalingam et al. [39], which could be systematically injected and excreted by the renal system, thus enabling intraoperative ureteral identification without the need for ureteral stenting. However, both reports concern in animal studies, and they have not yet been tested in humans.

#### 3.2.2. Intraoperative Urethral Assessment

With the progress made in transanal surgery and especially transanal total mesorectal (TaTME) excision, one of the main complications reported is urethral injury. The reported rates of urethral injury in TaTME range from 1% [40] to 6.7% [41]. First, Barnes et al. described the successful use of ICG mixed with Instillagel^®^ (CliniMed, Buckinghamshire, UK) to visualize the male urethra in eight cadavers [42]. In another cadaver study, the same group demonstrated successful visualization of the male urethra with an ICG-silicone coated Foley catheter [43]. The authors also tested a new dye (IRDye800BK) in this same study, which has greater depth of penetration than ICG. Barberio et al. [44] demonstrated the efficacy of ICG/Instillagel^®^ coated Foleys in cadaveric models [43]. Nitta et al. [45] successfully utilized the IRIS U kit (Stryker, Kalamazoo, MI, USA), an infrared lighted ureteral stent, for urethral visualization in a TaTME case report [45]. Although it seems reasonable that the ICG infusion used for ureteral visualization should also aid in visualizing the urethra, currently there are no available literature reports regarding the use of this technique for urethral visualization during transanal surgery.

#### 3.2.3. Intraoperative Nerve Assessment

Intraoperative nerve identification is essential, especially in pelvic dissection, as it could potentially allow the surgeon to recognize and spare these anatomical structures from adverse urogenital and anorectal sequelae. Recently Jin et al. [46] reported a pilot trial in which ICG was used to guide intraoperative nerve identification in colorectal surgery. They injected 4.5 mg/kg ICG intravenously 24 h before surgery in seven patients. Using this technique, the authors confirmed the intraoperative identification of the inferior mesenteric artery plexus and sacral plexus. Their trial was the first to report ICG use for identifying pelvic nerves in colorectal surgery. However, further studies are needed to define the optimal dosage and administration time of ICG.

### 3.3. Tumor Assessment

Tumor visualization and localization can be beneficial in many scenarios of colorectal surgery. There can be discrepancies in tumor location between endoscopic assessment and intraoperative findings; this sometimes results in a different surgical strategy to the one originally planned. In addition, tumor visualization may sometimes allow the surgeon to better delineate the resection margins. The first report of intraoperative fluorescent tumor localization was published in 2016 by Handgraaf et al. [47], although the main focus was sentinel lymph node identification using ICG. There are several reports in the literature [48,49,50,51,52] regarding the feasibility of ICG imaging in primary tumor visualization and real-time navigation. Atallah et al. [53] reported a case wherein fluorescence-guided surgery along with a robotic-assisted stereotactic navigational system were successfully utilized for complex locally advanced rectal cancer surgery. More recently, Satoyoshi et al. [54] reported a case series wherein ICG was injected near the tumor in the submucosal layer of the bowel preoperatively using endoscopy. The tumor detection rate was 100% within six days of preoperative marking, and the ICG did not cause any inflammatory phenomena (compared to conventional preoperative tattooing) [54]. A similar later study by Ahn et al. [55] confirmed this finding but further focused on better lymph node visualization and retrieval using ICG. Another novel fluorescent adjunct for intraoperative tumor localization was presented by Narihiro et al. [56]. They reported the use of a novel fluorescent clip (ZEOCLIP FS, Zeon Medical Co, Tokyo, Japan), which was placed a few days prior to the surgery and was easily detected from the serosal side intraoperatively. Similar clips were more recently reported by Lee et al. [57] and Ryu et al. [58].

### 3.4. Lymphatic Mapping

Colorectal cancer spreads through the lumen to the submucosa, where it can start spreading through the lymph nodes. Thus, lymph node yield is critical, and the removal of all potential tumor involved lymph nodes is mandatory. Nishigori et al. [59] performed perioperative, peritumoral, and submucosal ICG injection in 21 patients. They noted that lymph flow was intraoperatively visible in 18/21 patients. Most importantly, they reported a change in the extent of mesenteric resection and vessel ligation in 23.5% of patients. Similar results are reported in later studies [54,60,61,62]. Along with the feasibility of the intraoperative ICG imaging of lymphovascular drainage in colorectal cancer, a later study by Chand et al. [63] also showed that the success of lymphatic imaging was affected by the quantity of ICG injected into the bowel wall and that India ink tattooing that could potentially block lymph drainage and cause inflammatory phenomena. Another study by Goo et al. [64] that included 1079 patients reported that the intraoperative ICG imaging of lymph drainage, although useful in adequate lymph node yield in early colorectal cancers, did not yield the same results for more advanced stages. This result may be explained by the later study of Kakizoe et al. [65]. The authors performed a histopathologic study on splenic flexure cancer specimens to compare the association of fluorescence and cancer spread. They reported that the lymph nodes with cancer did not show any fluorescence, while 80% of the non-occupied lymph nodes were visible with ICG imaging. This could be a downside to the surgery’s lymphatic mapping and strategic planning since tumor metastasis might ‘silence’ the lymph nodes and lymphatic drainage that need to be included in the resected specimen. Similar results by Ushijima et al. [66] indicated that the ICG imaging of lymph flow was effective in early and not advanced colorectal cancer in their series of patients.

The idea of the better visualization of the lymphatic flow led to the utilization of intraoperative ICG imaging to perform CME and right hemicolectomies with D3 lymphadenectomy [67,68,69,70]. A recent matched control study by Park et al. involving patient with right-sided T3 and T4 colon cancer, showed that although ICG-guided lymphadenectomy resulted in a significantly higher lymph node yield (39 vs. 30, *p* = 0.003), the number of metastatic lymph nodes was not significantly different between the two groups [71].

More recently, the interim analysis of the GREENLIGHT trial, [72] a prospective non-randomized trial of ICG lymphatic mapping, showed that the extent of the lymphadenectomy was modified based on the results of imaging in 50% of the study population (35/70). What was more interesting is that they demonstrated that lymph node metastasis was not correlated with a paucity in ICG signal, as was shown in previous studies. In fact, one third of the fluorescent resected D3 lymph nodes were positive for malignancy.

### 3.5. Sentinel Lymph Node Assessment

**Figure 4:**
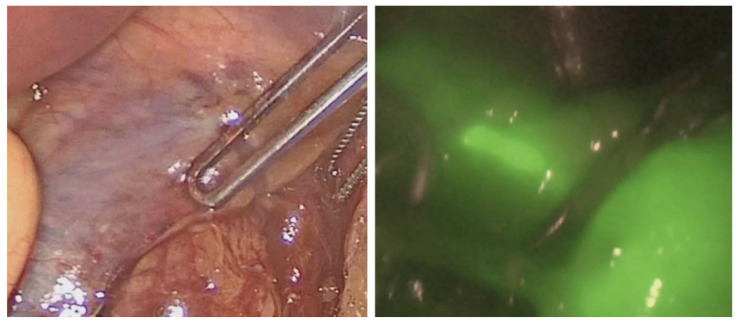
Sentinel lymph node identification using white light (**left**) and indocyanine green imaging (**right**). Courtesy of the International Society for Fluorescence Guided Surgery (ISFGS).

An early systematic review and meta-analysis [73] that included 52 studies (*n* = 3767 patients) concluded that the sentinel node biopsy was sensitive regardless of tumor location and T stage The role of the sentinel node in lymph node metastasis detection was initiated by Gould et al. in 1960 [74]. Although it is well established in plastic, breast, and ENT surgery, there is no such consensus in colorectal surgery. Additional studies [75,76,77,78,79,80,81,82,83] have since been published on the matter, specifically related to techniques of detecting sentinel lymph nodes in colorectal cancer, such as ICG (Figure 4). Since then, even more systematic reviews and metanalyses on this subject have been published [84,85]. The most recent was published in 2021 [86] and the reported pooled detection and accuracy rates were 90% and 77%, respectively, for the T3–T34 and 91% and 98%, respectively, for the T1–T2 group using the ICG technique for sentinel lymph node assessment. Sensitivity and accuracy were 2.31 [CI: 1.15–4.67] and 1.25 [CI 1.05–1.47] times higher in the T1–T2 group than the T3–T4 group. A more recent study by Picchetto et al. [87] examined the use of ex vivo sentinel lymph node assessment, reporting promising and reliable results in 22 patients.

### 3.6. Lateral Pelvic Lymph Node Assessment

The role of lateral pelvic node dissection (LPND) in locally advanced rectal cancer is still controversial, with results from recent meta-analyses stating that it does not affect disease-free or overall survival but may reduce local recurrence risk [88,89]. Nevertheless, this procedure is associated with longer operative times, higher morbidity rates than TME [88,89], and poor visualization due to complex anatomy. In this context, there have been some recent reports [90,91,92,93,94,95,96,97] on the utilization of ICG for intraoperative visualization during lateral lymph node dissection for locally advanced rectal cancer. Zhou et al. [90] compared two patient cohorts undergoing LPND, one under ICG-guidance and a standard one and showed that using ICG led to significantly lower blood loss and higher lymph node yield. In a propensity score-matched analysis, Dai et al. [92] confirmed the previous results that ICG-guided LPND leads to significantly higher lymph node yield and lower intraoperative blood loss. This group also reported shorter hospital stays and lower rates of residual disease/recurrence in the ICG group. Another study by Yasui et al. [93] reported using ICG to identify the SNL in LPND. All 21 patients included in the study underwent SNL identification before laparoscopic LPND. All patients with negative SNL had no disease in the remaining lymph nodes retrieved [93]. The same concept had been previously supported by Noura et al. [82]. Another more recent propensity match analysis involving 172 patients showed that using ICG in LPND results in a significantly higher lymph node yield than the conventional method (14 vs. 9 lymph nodes), with no significant differences in short-term complications [94]. The use of ICG for LPND needs to be fully established, since there is still no solid evidence of the clear benefit of this procedure in terms of survival [95]. Finally, in 2020, Kim et al. [96] reported a new method combining ICG lateral pelvic lymph node identification with 3D reconstruction images using the results of preoperative CT scans. More research is needed to elucidate any potential value of LPDN for locally advanced rectal cancer. The results and perspectives of ICG-guidance, given the complexity and morbidity associated with LPDN, are promising.

### 3.7. Distant Disease Assessment

#### 3.7.1. Peritoneal Metastases Assessment

The intraoperative detection of peritoneal metastases, even those not visible to the naked eye, is a great tool not only for diagnosis but also for treatment in select patients undergoing cytoreduction and intraperitoneal hyperthermic chemotherapy. First, Liberale et al. [97] reported the feasibility of ICG imaging in non-mucinous colorectal peritoneal metastases. They also reported a change in the surgery plan in 1/3 of the study population as ICG made visible peritoneal lesions that could not be seen with the naked eye [97]. A relevant metanalysis [98] published in 2020 included three studies and a total of 28 patients on the ICG imaging of colorectal cancer peritoneal metastases [97,99,100]. In all patients, ICG was intravenously administered between 0–24 h before surgery. The sensitivity of the method varied between 72.4% and 96.9%, while the specificity varied between 60% and 100%. Following this, in 2022, González-Abós et al. [101] reported preliminary results of the ICCP study, a prospective single center trial aiming to assess the diagnostic accuracy of the quantitative ICG imaging of non-mucinous colorectal peritoneal metastases. The authors reported that ICG uptake <100 units may suggest benign pathology while >181 units is suggestive of malignancy (sensitivity 89% and specificity 85%). The jury is still out as to whether this modality could be the standard of care in diagnosing/treating this specific category of patients with peritoneal metastases.

#### 3.7.2. Liver Metastases Assessment

Colorectal liver metastases are treatable with resection. However, the indications for the hepatic metastasectomy of colorectal origin are still evolving. Nevertheless, up to 1/3 of patients undergoing the curative resection of colorectal hepatic metastases will have R1 resections (<1 mm resection margin) or residual disease due to the inability to properly detect intraoperatively [102]. This can severely impact disease-free survival and overall survival. The intraoperative imaging of the metastases is mostly performed using visual and haptic feedback and intraoperative ultrasound. There have been several reports on the use of ICG for intraoperatively localizing colorectal liver metastasis. The most recent review was by Picollo et al. [102] in 2022 and included 13 studies (literature search through April 2021). The authors reported a wide variation in the timing of ICG administration (1 to 14 days preoperatively) as well an average detection rate of metastases with ICG of 79.03% (range: 57.6–100) compared to 95.97% (range: 93.3–100%) with intraoperative ultrasound. Since then, four more studies have been published on this issue. In 2021, Picollo et al. [103] reported the advantage and the possible replacement of tactile feedback with ICG imaging, a potentially important feature, especially during minimally invasive surgery. Nevertheless, this study also included patients with hepatocellular carcinoma and cholangiocarcinoma, therefore, the results cannot be generalized. A prospective single-center UK study published in 2022 [104] included 15 patients undergoing ICG-assisted liver metastasectomy. The authors reported that ICG altered the operative plan in almost half of the patients (43%), despite the concurrent use of intraoperative ultrasound. Finally, a randomized control trial by He et al. [105] involving 64 patients undergoing hepatic metastasectomy for colorectal cancer showed that the mean number of intraoperatively identified colorectal metastases was significantly higher (3.03, SD:1.58) in the ICG imaging group compared to the non-ICG group (2.28 SD:1.35). The authors also noted that the 1-year recurrence rate was significantly lower in the ICG group, while postoperative complications were comparable between the two groups. Nevertheless, imaging with ICG, despite minimizing the need for tactile feedback, can be non-specific, especially regarding the resection margins. In this scope, Nishino et al. [106] recently published an animal study on the ICG-guided resection of colorectal metastases in mice previously injected with a carcinoembryonic antigen antibody conjugated with a fluorophore. At three weeks after surgery, tumor weight was significantly lower in the ICG imaging group compared to the control group. The authors concluded that this technique might potentially improve hepatic metastasectomy [106].

## 4. Discussions and Future Perspectives

ICG in colorectal surgery can be beneficial in multiple ways (Table 1). Although it has been FDA-approved since 1959, [1] the use of ICG has become more prominent in colorectal surgery over the past ten years. Still, it is still not routinely adopted by colorectal surgeons as some question its real benefit due to the lack of high-level evidence. In addition, some have also raised the issue of additional costs of ICG without a scientifically proven benefit. Nevertheless, a recent cost analysis [20] on the routine use of ICG imaging for anastomotic perfusion assessment reported that it is cost effective. Based on the assumption that the cost of AL is more than USD 5616.29 and the cost of ICG-FA is <USD 634.44, and that the given odds ratio for AL reduction with ICG-use (based on a literature review) is 0.46, the authors showed that routine use of ICG for anastomotic assessment can reduce the financial burden of AL.

ICG is a safe and relatively inexpensive method of perfusion assessment; current data show its beneficial role in reducing AL. Nevertheless, this is yet to be proven in well-conducted and powered randomized control trials. Hopefully, the results of the AVOID study will provide the evidence needed to dissolve any ensuing skepticism on this modality [15]. A recently published Delphi consensus of international experts on the use of ICG further supported the use of ICG for anastomotic perfusion assessment (100% consensus reached), as well as issues such as timing and dosage of ICG for perfusion assessment during colorectal surgery [108,109]. The other ICG applications for perfusion assessment, including for gracilis and advancement flaps, although promising, require further research in order to be routinely implemented into daily practice.

The available studies show promising results with potential practice-changing applications of this technique including lymph node evaluation. The prematurity and contradictive nature of the available data is also reflected in the recent Delphi survey: while experts agreed that ICG lymphangiography might increase the lymph node yield, they debated whether the routine use of ICG is needed in cancer surgery for lymph node evaluation or whether it will impact the resection plan [108,109]. Furthermore, the use of ICG for the intraoperative visualization of colorectal peritoneal and liver metastases seems revolutionary, yet more research is needed for the quantification of ICG signal as well as safely defining the boundary where fluorescence ceases and healthy tissue ensues. Finally, intraoperative ICG ureteral visualization, although helpful especially in complicated cases, still lacks the necessary evidence for routinely implementing this technique in everyday practice. The parameters listed in Table 2 need to be considered when ICG is utilized for bowel perfusion assessment and lymph node evaluation.

The future may be a combination of ICG applications optimized by artificial intelligence (AI) programs. In 2020, Park et al. [110] reported on an AI/ICG based real-time micro perfusion application that was more accurate and consistent than conventional ICG imaging. A similar hybrid model was reported by Seeliger et al. [111] The authors performed a quantitative mucosal and serosal perfusion analysis in porcine ischemic colons using ICG and computer assisted FLER analysis. They suggested that serosal ICG assessment might be less indicative of the actual extent of ischemia than the mucosal ICG assessment. These are only some examples of the evolving AI technology in ICG imaging studies with potential applications in better quantifying ICG signal and delineating tumor location and resection margins for colorectal metastases.

## 5. Conclusions

ICG imaging is undoubtedly a helpful adjunct. ICG perfusion assessment seems to reduce AL. Its use in ureteric identification, lymphatic mapping and assessing liver and peritoneal metastases remain developmental. To date, these are the reported applications of ICG in colorectal surgery. Evidence is low for the majority of these applications.

## Figures and Tables

**Table 1:** Reported applications of indocyanine green (ICG) imaging in colorectal surgery in literature.

ICG Applications in Colorectal Surgery
Perfusion Assessment
Bowel Perfusion [5,6,7,8,9,10,11,12,13,14,15,16,17,18,19,20]
Anastomotic Perfusion [5,6,7,8,9,10,11,12,13,14,15,16,17,18,19,20,26,27,28,29]
Gracilis Muscle Perfusion [24]
Anal Advancement Flap Perfusion [25]
Pedicled Omentoplasty [21,22]
Anatomic Visualization
Ureteral Visualization [31,32,33,34,35,36,38,39]
Urethral Visualization [42,43,44,45]
Nerve assessment [46]
Tumor Localization [47,48,49,50,51,52,53,54,55,56,57,58]
Lymphatic Mapping [59,60,61,62,63,64,65,66,67,68,69,70,71,72]
Sentinel Lymph Node Identification [75,76,77,78,79,80,81,82,83,84,85]
Lateral pelvic node dissection [88,89,90,91,92,93,94,95]
Distant Metastases Assessment
Peritoneal Metastases [96,97,98,99,100,101]
Liver Metastases [101,102,103,104,105,106,107]

**Table 2:** Important parameters during evaluation of blood flow and lymphatic mapping using ICG.

Important Parameters during Evaluation of Blood Flow and Lymphatic Mapping Using ICG
Dosage of ICG
Concentration of ICG
Route of administration
Assessment time (time from ICG injection until assessment)
Quantification of the result

## Data Availability

Not applicable.

## Supplementary Materials

The following supporting information can be downloaded at: https://www.mdpi.com/article/10.3390/jcm12020494/s1, Video S1: demonstrates the intraoperative use of ICG for bowel perfusion assessment.
